# Evidence for Anger Saliency during the Recognition of Chimeric Facial Expressions of Emotions in Underage Ebola Survivors

**DOI:** 10.3389/fpsyg.2017.01026

**Published:** 2017-06-23

**Authors:** Martina Ardizzi, Valentina Evangelista, Francesca Ferroni, Maria A. Umiltà, Roberto Ravera, Vittorio Gallese

**Affiliations:** ^1^Department of Medicine and Surgery, Unit of Neuroscience, University of ParmaParma, Italy; ^2^Ravera Children Rehabilitation CentreFreetown, Sierra Leone; ^3^Department of Food and Drug Sciences, University of ParmaParma, Italy; ^4^Department of Health Psychology, ASL 1 (Azienda Sanitaria Locale) ImperieseSanremo, Italy; ^5^Institute of Philosophy, School of Advanced Study, University of LondonLondon, United Kingdom

**Keywords:** anger, childhood trauma, chimeric facial expressions, Ebola, emotions, eye, mouth, recognition bias

## Abstract

One of the crucial features defining basic emotions and their prototypical facial expressions is their value for survival. Childhood traumatic experiences affect the effective recognition of facial expressions of negative emotions, normally allowing the recruitment of adequate behavioral responses to environmental threats. Specifically, anger becomes an extraordinarily salient stimulus unbalancing victims’ recognition of negative emotions. Despite the plethora of studies on this topic, to date, it is not clear whether this phenomenon reflects an overall response tendency toward anger recognition or a selective proneness to the salience of specific facial expressive cues of anger after trauma exposure. To address this issue, a group of underage Sierra Leonean Ebola virus disease survivors (mean age 15.40 years, SE 0.35; years of schooling 8.8 years, SE 0.46; 14 males) and a control group (mean age 14.55, SE 0.30; years of schooling 8.07 years, SE 0.30, 15 males) performed a forced-choice chimeric facial expressions recognition task. The chimeric facial expressions were obtained pairing upper and lower half faces of two different negative emotions (selected from anger, fear and sadness for a total of six different combinations). Overall, results showed that upper facial expressive cues were more salient than lower facial expressive cues. This priority was lost among Ebola virus disease survivors for the chimeric facial expressions of anger. In this case, differently from controls, Ebola virus disease survivors recognized anger regardless of the upper or lower position of the facial expressive cues of this emotion. The present results demonstrate that victims’ performance in the recognition of the facial expression of anger does not reflect an overall response tendency toward anger recognition, but rather the specific greater salience of facial expressive cues of anger. Furthermore, the present results show that traumatic experiences deeply modify the perceptual analysis of philogenetically old behavioral patterns like the facial expressions of emotions.

## Introduction

Traumatic childhood experiences alter victims’ emotional development, with a negative impact on affect recognition, social interactions and self-regulation abilities. Exposure to early adverse experiences produces chronic and specific shifts in the explicit recognition of the facial expressions of negative emotions, generating a severe bias in the recognition of anger. Even if this phenomenon has been established among several samples exposed to disparate traumatic experiences and by means of different experimental procedures, to date it remains unclear if it can be associated to an overall response tendency toward anger recognition or to the greater perceptual salience attributed to the facial expressive cues of anger after trauma exposure. In this study, the mechanism underlying the bias in the recognition of angry facial expressions was investigated in Sierra Leonean adolescents exposed to the recent Ebola virus outbreak and in a local population of age-matched controls through a forced-choice recognition task of chimeric facial expressions of emotions.

Ebola virus gained widespread attention in the fall of 2014 when West Africa was plagued by the largest Ebola outbreak reported in history. Ebola virus is transmitted by direct and indirect contact with blood, feces, or body fluids from an infected person or by direct contact with the virus, as in a laboratory. The incubation period ranges from 2 to 21 days. Death can occur within 10 days from symptoms onset. The average Ebola Virus Disease (EVD) case fatality rate is around 50%, with a range from 25 to 90% in past outbreaks. Currently, patients receive supportive and symptomatic therapy, since there is no specific treatment for the disease. West Africa countries mainly affected by Ebola outbreak were Guinea, Liberia and Sierra Leone where 28610 confirmed, probable, and suspected cases have been reported, with 11308 deaths since the onset of the Ebola outbreak (World Health Organization, 2016). The majority of these cases and deaths were reported between August and December 2014, date after which case incidence began to decline thanks to the scale-up of treatment, isolation, and safe burial practice in the three countries.

Specifically, in Sierra Leone the Ebola outbreak affected every district of the country. A national state of emergency was declared on July 31st, 2014; the closure of all schools, the institution of free Ebola zones controlled by check-points and the quarantine of affected communities were the measures put in place to stop the spread of the deadly virus. According to the World Health Organization (2016), a total of 14122 clinical cases of Ebola have been recorded (8704 confirmed) with a total of 3955 deaths in Sierra Leone. Considering underage Sierra Leonean population, a recent report identified 12023 children orphaned by Ebola across the country and the possible existence of additional 3630 children who have not yet been identified, either because of remoteness or because they lost their parents after the report data collection (Street Child of Sierra Leone, 2015).

This concise survey shows that underage Ebola survivors were exposed to extraordinary traumatic events: the risk of death due to the disease, the possible loss of their primary caregivers or relatives, and the following neglect, stigma, malnutrition and lack of access to education.

Empirical interest in the psychological effects of trauma on underage victims led the proliferation of studies about the consequences of the exposure during childhood to traumatic events like natural disasters, terrorist attacks, armed conflicts, health emergencies, as well as, physical and sexual abuses and neglect. From the recent literature, it has been demonstrated that adverse, maltreating, neglectful, and physically abusive developmental environments are associated with specific changes in victims’ ability to explicitly recognize emotional signals like facial expressions of emotions (da Silva Ferreira et al., 2014). This is particularly relevant because it means that early traumatic experiences are able to deeply modify the processing of phylogenetically old behavioral patterns like the facial expression of emotions. Indeed, according to Basic Emotion Theory (BET) (Ekman and Friesen, 1969), the so-called basic emotions (i.e., fear, disgust, anger, joy, sadness, and surprise) are considered as response-coordination packages, associated with characteristic configurations of facial muscle movements sculpted by evolution to meet particular environmental challenges, such as avoiding environmental threatening (Ekman, 1992).

The most common consequence of trauma exposure on victims’ ability to recognize facial expressions of emotions is the development of an explicit recognition bias for the facial expression of anger. Specifically, when victims of maltreatment and neglect are forced to explicitly identify negative facial expressions of emotions (e.g., fear and sadness) they preferentially recognize them as anger (Ardizzi et al., 2013, 2015). Maltreated children also showed an overall response tendency for anger, identifying this emotion more frequently than controls (Ardizzi et al., 2015). Similar results were found by Pollak et al. (2000) among physically abused children, who set a lower threshold for selecting angry faces than did their non-abused peers, demonstrating an overall bias toward facial expressions of anger.

Differently, in a subsequent study Pollak and Sinha (2002) evidenced that even if physically abused children recognized angry facial expressions on the basis of less sensory inputs with respect to controls, when they had to identify highly degraded facial expressions of emotions, they did not show an overall anger response tendency (Pollak and Sinha, 2002). These authors suggested that physically abused children’s recognition of facial expressions is guided by their perceptual sensitivity for angry expressive cues rather than by an overall response bias. Coherently with this suggestion, when severely abused children were asked to identify emotional faces that had been morphed with different emotions (e.g., sadness to anger) they selectively over-identified anger only when discriminating angry faces morphed with either fearful or sad facial expressions (Pollak and Kistler, 2002). Using similar mixed morphed facial expressions, a biased pattern of facial expressions recognition has been described also among children who experienced a single life-threatening event like a terrorist attack (Scrimin et al., 2009). Additionally, a subsequent study conducted among traumatized children, supported the greater perceptual salience of angry facial expressive cues, demonstrating an earlier identification of facial expressions of anger even when they morphed from neutrality to the peak of emotion (Pollak et al., 2009).

This plethora of studies supports two different interpretations of the explicit recognition bias for the facial expression of anger. On one hand, this phenomenon can reflect an overall response tendency toward anger recognition (Pollak et al., 2000; Ardizzi et al., 2013, 2015). On the other, the explicit recognition bias for the facial expression of anger can be related to the increased perceptual salience of specific facial expressive cues of anger (e.g., eye-region or mouth-region) after trauma exposure (Pollak and Kistler, 2002; Pollak and Sinha, 2002; Pollak et al., 2009; Scrimin et al., 2009).

To disentangle between these two different hypotheses, a group of underage Sierra Leonean Ebola survivors and an age-matched control group performed a forced-choice recognition task of chimeric facial expressions of emotions. The chimeric facial expressions were obtained pairing upper and lower half faces of two different negative emotions (selected from anger, fear and sadness, for a total of six different combinations).

To decode facial affects both configural and part-based information processes are involved (Calder et al., 2000). Facial expressions of basic emotions are produced with characteristic configurations of facial muscle movements that provide the perceptual basis for discriminating between distinct types of emotional expressions (Ekman and Friesen, 1977). Configurational processing refers to the role of multiple facial features and their inter-relationship in facial expressions recognition and judgment. An illustrative example is the well-known Thatcher illusion, according to which the judged pleasantness of upright and inverted smiling mouths is affected by irrelevant facial features like the location of the eyes in relation to the mouth (above or below), and the distance between the eyes and the mouth (Parks et al., 1985). Pairing upper and lower half faces of two different facial expressions displayed by the same actor create a perceptually “new composite” facial expression in which the two parts interact during an explicit recognition of the emotion (Calder et al., 2000). When a forced-choice identification judgment on the chimeric facial expressions is required, part-based information processes arise, revealing the predominance of one of the two half faces over the other (Dunlap, 1927). In other words, even if facial expressions can be considered an “holistic object,” different facial areas are more responsible for the recognition of different facial expressions (Adolphs, 2002). Investigations on this issue revealed that some facial expressions of emotions are more readily recognizable from the upper face region (i.e., anger, fear, and sadness), whereas others are more readily identified from the lower face region (i.e., happiness and disgust) (Hanawalt, 1944; Bassili, 1979; Ekman, 1982; Smith et al., 2005; Nusseck et al., 2008; Eisenbarth and Alpers, 2011; Blais, 2012; Schurgin et al., 2014).

In the present study we took advantage from these perceptual processes involved in the processing of chimeric faces expressing emotions to investigate the mechanisms underlying the well know bias in the recognition of facial expressions of anger.

We used three facial expressions of negative emotions (i.e., anger, fear, and sadness) to compose the chimeras, first because the behavioral bias in the recognition of facial expressions of anger was visible mainly when victims of trauma were judging negative facial expressions. Second, because according to the literature all these three emotions are better recognized from the upper face. Starting from these assumptions, we expected to find an overall identification performance mainly guided from the eye-cue, confirming the presence of part-based information processing in the recognition of chimeric facial expressions of negative emotions.

Considering the main aim of the present study, if the explicit recognition bias for facial expressions of anger reflects a specific greater salience of angry facial expressive cues to the detriment of other emotions’ expressive cues, Ebola survivors’ part-based chimeric facial expressions identification should be driven by the presence of facial expressive cues of anger regardless of their position.

Differently, if the bias in the recognition of facial expressions of anger is the outcome of a mere response tendency toward anger recognition, a general propensity to recognize the facial expressions anger in Ebola survivors might be expected regardless of the emotions composing the chimeric facial expressions.

## Materials and Methods

### Participants

Sixty-one underage Sierra Leonean participants were recruited for the study. Of these 30 were EVD survivors (S-group: mean age 15.40 years, SE 0.35; years of schooling 8.8 years, SE 0.46; 14 males) and 31 were controls (C-group: mean age 14.55, SE 0.30; years of schooling 8.07 years, SE 0.30, 15 males). No significant between-groups difference was estimated for age (*t*_59_ = 1.84, *p* = 0.07) and years of schooling (*t*_58_ = 1.27, *p* = 0.21). The sample size exceeded the minimum amount required (*n*. 56) estimated by means of statistical power analysis (a priori sample size n. evaluated for 1-β = 0.95, α = 0.05 and effect size = 0.25). The sampling was suspended when two gender-balanced groups of enough size were obtained.

Participation in the study was completely voluntary, no participant has been repaid. Participants were recruited with the support of non-profit organizations (RCRC and FHM-Italia Onlus) working with Sierra Leonean youths. S-group participants came from Freetown East area that was the most affected by EVD. They were selected on the basis of medical records describing the date of Ebola infection, medical treatments received and recovery date. On average EVD infection lasted 22.7 days (SE 3.39) and was contracted 230 (SE 19.51) days before the execution of the study. All S-group participants were hospitalized for an average period of 296.9 days (SE 27.13) and lost, on average, 4.6 (SE 0.71) family members as a consequence of EVD. After EVD recovery, all S-group participants described stigmatization and exclusion episodes, a reduction of access to education and work opportunities. C-group participants were recruited among people from Freetown but resettled in neighboring villages monitored by checkpoints during the Ebola outbreak. As a consequence of this practice for infection prevention and control, none of C-group participants contracted EVD and lost family members.

### Questionnaires and Scales

Participants’ demographic data (i.e., gender, age, weight, height, dominant hand, level and years of schooling, first and second language, ethnic group), medical and pharmacological information about actual and past health conditions (i.e., disease duration, sanitary treatments, hospitalization, family members infected and deceased), participants’ socio-economic status (i.e., family unit, members of household, occupation) and critical life events (i.e., sexual violence, physical violence, abuse, maltreatment) were collected by means of ad-hoc designed interviews. Partial or unclear information was completed and checked thanks to sanitary, educational or charitable institutions. Furthermore, self-perceived risk (P.R) and comparative perceived risk (P.C.R) about common infective and metabolic diseases were assessed. Participants were asked to evaluate the probability to contract different illnesses both in the next 12 months (P.R.) and with respect to western age- and gender-matched people (P.C.R). To exclude the presence of visual deficits, participants’ visual acuity (20/20) was estimated following standard procedure by means of Snellen chart (Snellen, 1862). Moreover, kinetic visual field test and pupillary light response (i.e., direct and consensual light reflexes) tests were conducted.

In order to evaluate participants’ cognitive performance and naming skills, Standard Progressive Matrices test (SPM, Raven et al., 1998) and Boston naming test (BNT; Kaplan et al., 1983) were administered. No significant difference was found between the two groups for BNT (S-group: 21.70, SE 1.05; C-group: 22.97, SE 0.93; *t*_59_ = -0.90, *p* = 0.37) and SPM scores (S-group: 75.74, *SE* = 2.81; C-group: 72.62, *SE* = 1.04; *t*_46_ = 1.20, *p* = 0.24).

Moreover, three clinical scales commonly used to evaluate the psychological impact of negative events and the presence of symptoms of post-traumatic stress disorders were submitted to a cross-cultural adaptation process, translated from English to Krio and tested in an independent Sierra Leonean sample. The Guidelines for the Process of Cross-Cultural Adaptation of Self-Report Measures (Beaton et al., 2000; World Health Organization, 2010) were followed for the translation of the scales. For a detailed description of the Cross-Cultural Adaptation procedure followed please, see the Supplementary Data and Table 1. The PTSD Checklist for DSM-5 (PCL-5, Weathers et al., 2013) is a 20-item self-report measure assessing the 20 DSM-V symptoms of PTSD. The Impact of Event Scale-revised (IES-R, Weiss and Marmar, 1996) is a 22-item self-report measure that assesses subjective distress caused by traumatic events. The items correspond directly to 14 DSM-IV symptoms of PTSD. Respondents were asked to identify a specific stressful life event and then indicate how much they were distressed or bothered during the past 7 days by each “difficulty” listed. To the purposes of the present study, all participants were asked to answer by considering the Ebola outbreak as the stressful life event. The Cognitive Emotion Regulation Questionnaire short version (CERQ-short, Garnefski and Kraaij, 2006) is a 18-item self report scale evaluating the role played by victims’ emotion regulation in adaptation to stressful life events.

Interestingly, S-group’s total scores obtained at PCL-5 (*t*_59_ = 4.42, *p* = 0.00) and IES-R (*t*_59_ = 5.68, *p* = 0.00) scales resulted significantly higher with respect to C-group’s scores. Furthermore, S-group showed a significant higher incidence of rumination (*t*_59_ = 3.52; *p* = 0.001) and catastrophizing (*t*_59_ = 6.25; *p* = 0.000) tendencies but also a significant greater refocus on planning (*t*_59_ = 6.97; *p* = 0.000) and positive reappraisal (*t*_59_ = 3.61; *p* = 0.001) coping strategies with respect to C-group. See **Table 1** for mean scores and significant differences between the two groups in PCL-5, IES-R and CERQ-short subscales.

**Table 1:** Questionnaire scores of Ebola Virus disease Survivors (S-group) and Controls (C-group).

Scales	Subscales	S-group	C-group	Between-groups differences
PR		1.43; SE 0.078	1.35; SE 0.031	*t*_59_ = 1; *p* = 0.320
CPR		4.19; SE 0.11	4.15; SE 0.061	*t*_59_ = 0.37; *p* = 0.712
BNT		21.70; SE 1.05	22.97; SE 0.93	*t*_59_ = -0.90; *p* = 0.370
SPM		75.74; SE 2.81	72.62; SE 1.04	*t*_46_ = -1.20; *p* = 0.240
IES-R_TOT^∗^		1.63; SE 0.09	0.65; SE 0.14	*t*_59_ = 5.68; *p* = 0.000
	Intrusion^∗∗^	1.50; SE 0.10	0.58; SE 0.13	*t*_59_ = 5.49; *p* = 0.000
	Avoidance^∗∗^	1.89; SE 0.10	0.74; SE 0.16	*t*_59_ = 5.97; *p* = 0.000
	Hyperarousal^∗∗^	1.44; SE 0.12	0.63; SE 0.16	*t*_59_ = 4.02; *p* = 0.000
PCL5_TOT^∗^		30.43; SE 2.01	16.55; SE 2.40	*t*_59_ = 4.42; *p* = 0.000
	Cluster B^∗∗∗^	9.07; SE 0.80	4.55; SE 0.75	*t*_59_ = 4.13; *p* = 0.000
	Cluster C^∗∗∗^	3.37; SE 0.32	1.58; SE 0.38	*t*_59_ = 3.61; *p* = 0.001
	Cluster D^∗∗∗^	9.97; SE 0.91	5.90; SE 0.90	*t*_59_ = 3.17; *p* = 0.002
	Cluster E^∗∗∗^	8.03; SE 0.82	4.52; SE 0.72	*t*_59_ = 3.24; *p* = 0.002
	Pos. Prov. Diagnosis^∗^	50%	22.6%	*X*_(1)_ = 4.97; *p* = 0.026
CERQ-short				
	Self blame	2.53; SE 0.15	3.42; SE 0.31	*t*_59_ = -2.52; *p* = 0.014
	Acceptance	4.37; SE 0.26	4.00; SE 0.38	*t*_59_ = 0.79; *p* = 0.43
	Rumination^∗∗∗∗^	5.90; SE 0.35	4.13; SE 0.36	*t*_59_ = 3.52; *p* = 0.001
	Positive refocusing	6.63; SE 0.31	5.13; SE 0.47	*t*_59_ = 2.65; *p* = 0.01
	Refocus on planning^∗∗∗∗^	7.90; SE 0.25	4.58; SE 0.40	*t*_59_ = 6.97; *p* = 0.000
	Positive reappraisal^∗∗∗∗^	7.20; SE 0.33	5.16 SE 0.46	*t*_59_ = 3.61; *p* = 0.001
	Putting in to perspective	5.30; SE 0.33	4.45; SE 0.41	*t*_59_ = 1.60; *p* = 0.116
	Catastrophizing^∗∗∗∗^	7.50; SE 0.24	4.32 SE 0.44	*t*_59_ = 6.25; *p* = 0.000
	Other blame	3.00; SE 0.33	3.29; SE 0.28	*t*_59_ = -0.68; *p* = 0.502

*Significant between-groups differences were estimated. ^∗^*p* < 0.05, ^∗∗^Bonferroni corrected *p* < 0.016, ^∗∗∗^Bonferroni corrected *p* < 0.012, ^∗∗∗∗^Bonferroni corrected *p* < 0.005. PR, Self-perceived Risk; CPR, Comparative Perceived Risk; BNT, Boston Naming Test; SPM, Standard Progressive Matrices test; IES-R, Impact of Event Scale-revised; PCL-5, PTSD Checklist for DSM-5; Pos. Prov. Diagnosis, Positive Provisional Diagnosis; CERQ-short, Cognitive Emotion Regulation Questionnaire short version.*

### Procedure

Study general purposes and procedures were explained by local social-workers to volunteers and their legal guardians. After participants’ agreement in study involvement, a written informed consent was collected. The experimental protocol was approved by the Ministry of Health and Sanitation of the Republic of Sierra Leone and it was in line with the Declaration of Helsinki 2013.

The experimental session took place in a quiet room and consisted in a forced-choice recognition task of chimeric facial expressions of emotions. Participants were asked to identify adults’ chimeric facial expressions of emotions choosing one of the three proposed labels (i.e., anger, fear, and sadness).

**FIGURE 1:**
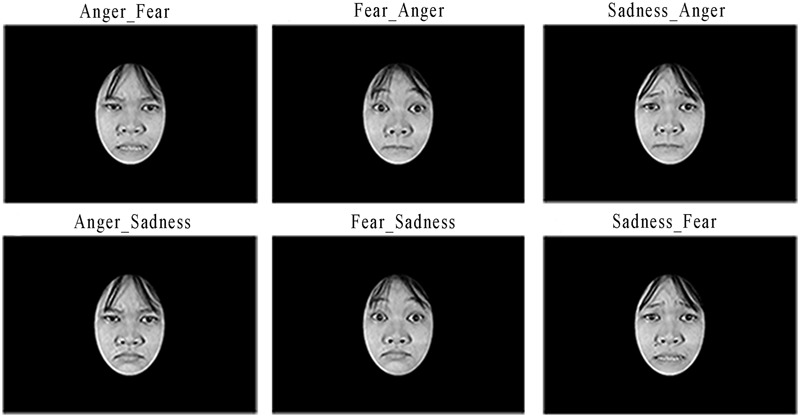
Exemplificative chimeric facial expressions employed in the present study.

Stimuli employed in this study were 96 images representing chimeric facial expressions obtained by pairing upper and lower half-faces of two different facial expressions of negative emotions. The gray scale images of facial expressions of negative emotions were acquired by the Montreal Set of Facial Displays of Emotion (Beaupré, 2005) that was already used in previous experiments conducted on Sierra Leonean population (Ardizzi et al., 2013, 2015, 2016; Umiltà et al., 2013). The Montreal Set of Facial Displays of Emotion images chosen to build the chimeric facial expressions were selected pseudo-randomly from the Asian, African, Hispanic and Caucasian sets, to include 16 instances of each of the chosen expressions (i.e., anger, fear, and sadness), balanced for gender and ethnic group. The chimeric stimuli were constructed by means of Adobe Photoshop CC2015 software. The upper (i.e., from forehead to the nose) and the lower (i.e., from the nose to the chin) half-faces, of the same actor, but showing different negative facial expressions, were combined creating a chimeric expression. The obtained chimeric expressions were resized at 800 pixels × 560 pixels and presented against a black background in an oval window. Six different combinations were obtained matching the upper and the lower half-faces of each selected negative emotion. The nomenclature of the six chimeric facial expressions adopted in the present study uses the initial of the negative emotion displayed in the upper half-face followed by the initial of the negative emotion showed in the lower half-face (i.e., AF: upper half-face = anger, lower half-face = fear; AS: upper half-face = anger, lower half-face = sadness; FA: upper half-face = fear, lower half-face = anger; FS: upper half-face = fear, lower half-face = sadness; SA: upper half-face = sadness, lower half-face = anger; SF: upper half-face = sadness, lower half-face = fear). For an explicative set of six chimeric facial expressions, please see **Figure 1**.

A local social-worker was always present to ensure that participants remained at ease, understood the instructions and to translate from English to Krio, whenever necessary. E-Prime 2.0 software (Psychology Software Tools, Inc.) was used for stimuli presentation.

After participants’ arrival, they filled the demographic interview, the validated questionnaires and the adapted clinical scales. During the forced-choice recognition task of chimeric facial expressions of emotions, participants sat comfortably at a table, in front of a computer monitor (1024 × 768@75 Hz). They were instructed to pay attention and to observe each stimulus for its entire duration. Each experimental trial started with the presentation of a centered cross for 1000 ms. Each stimulus, lasting 3000 ms, was displayed in random order (96 total trials, 16 trials for each of the 6 chimeric facial expressions). After each stimulus, with no time limit, participants were asked to identify which of the three alternative labels (i.e., anger, fear, and sadness) best described the chimeric facial expression displayed in the stimulus just shown. The three alternative labels were always visible and written in English and Krio. Participants’ forced-choice recognition performance could follow three different strategies. They could recognize the chimeric facial expression taking advantage from the facial cues displayed by the upper half-face (eye-cue driven response) or by the lower half-face (mouth-cue driven response). Being each chimeric facial expression composed by two of the three alternative negative facial expressions, participants could also choose a sort of “third way strategy” attributing to the chimeric facial expression the identity of the third facial expression not displayed neither in the upper nor in the lower half-faces (no-cue driven response).

The total duration of the forced-choice facial expressions recognition task was approximately 10 min, depending on participants’ response time.

## Results

Two participants were excluded from the analyses. One participant was excluded due to visual deficits incurred after EVD contraction and evidenced at the visual acuity examination. Another participant resulted outlier (2.5 SD) in task performance. The resulting sample consisted of 59 participants (28 S-group; 31 C-group). For a visual representation of the sample composition and selection, see the Supplementary Figure 1.

The recognition bias for facial expressions of anger was investigated conducting a repeated measure ANOVA on participants’ performance at the forced-choice recognition task of chimeric facial expressions of emotions. Group (S-group, C-group) was entered as between-factor; whereas Chimera (i.e., AF, AS, FA, FS, SA, and SF) and Cue (i.e., eye-cue, mouth-cue, and no-cue) as within-factors.

Mauchly’s test conducted indicated that the assumption of sphericity had been violated [Cue factor: χ^2^_(2)_ = 7.61, *p* = 0.02; Cue^∗^Chimera interaction: χ^2^_(54)_ = 233.56, *p* < 0.001], therefore df were adjusted using Greenhouse-Geisser correction (Cue factor: 𝜀 = 0.89; Cue^∗^Chimera interaction: 𝜀 = 0.54). The repeated measures ANOVA revealed that the factor Cue was significant (*F*_1.8,101.15_ = 76.86; *p* < 0.001; $\eta_{p}^{2}$ = 0.57), as well as the interactions Cue by Chimera (*F*_5.4,305.5_ = 31.02; *p* < 0.001; $\eta_{p}^{2}$ = 0.35) and Cue by Chimera by Group (*F*_10,570_ = 2.26; *p* < 0.014; $\eta_{p}^{2}$ = 0.04).

**FIGURE 2:**
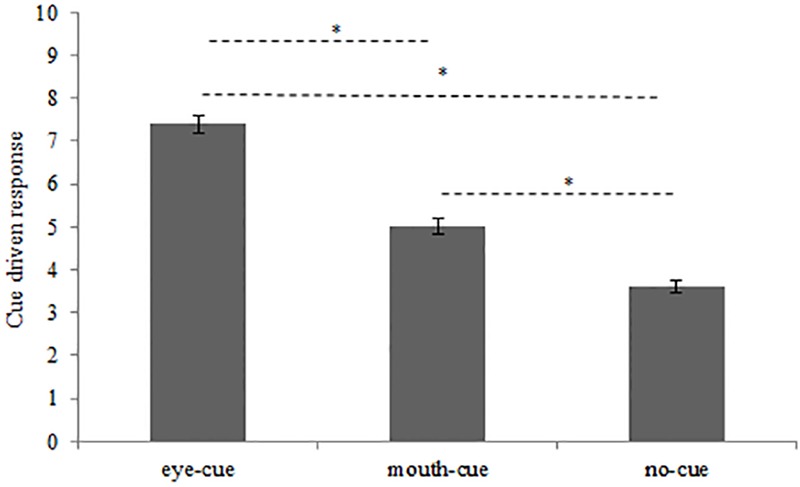
Number of chimeric facial expressions recognitions driven by eye-cue, mouth-cue, and no-cue. ^∗^*p* < 0.001; error bars depicted SE.

Sidak *post hoc* test conducted on Cue main effect (**Figure 2**) revealed that, regardless of participants’ group, the cue most used for chimeric facial expressions recognition was the eye-cue (7.39, SE 0.20) followed by mouth-cue (5, SE 0.19) and no-cue (3.60, SE 0.14). All comparisons resulted significantly different (all *p*_s_ < 0.001).

Sidak *post hoc* test conducted on the interaction Cue by Chimera demonstrated that considering the AF chimera, chimeric facial expressions recognition was significantly driven by no-cue, with no significant difference between eye-cue and mouth-cue (eye-cue: 4.27, SE 0.38; mouth-cue: 4.48, SE 0.41; no-cue: 6.84, SE 0.47; all *p*_s_ < 0.048). Concerning the AS chimera, the most used cue was the eye-cue resulting significantly different from both mouth-cue and no-cue (eye-cue: 9.30, SE 0.35; mouth-cue: 3.57, SE 0.28; no-cue: 3.12, SE 0.47; all *p*_s_ < 0.000). Regarding the FA chimera, the less used cue was no-cue with no significant difference between eye-cue and mouth-cue (eye-cue: 7.02, SE 0.40; mouth-cue: 6.89, SE 0.39; no-cue: 2.08, SE 0.29; all *p*_s_ < 0.000). Considering the FS chimera, the most used cue was eye-cue followed by no-cue and mouth cue. All comparisons were significant (eye-cue: 8.82, SE 0.48; mouth-cue: 2.78, SE 0.28; no-cue: 4.40, SE 0.41; all *p*_s_ < 0.009). Considering the SA chimera, again, all differences between cues were significant demonstrating that the most used cue was the mouth-cue, followed by eye-cue and no-cue (eye-cue: 5.13, SE 0.41; mouth-cue: 7.99, SE 0.39; no-cue: 2.81, SE 0.26; all *p*_s_ < 0.001). Lastly, considering the SF chimera, the most used cue was the eye-cue followed by mouth-cue and no-cue. All comparisons were significant (eye-cue: 9.83, SE 0.49; mouth-cue: 3.91, SE 0.42; no-cue: 2.26, SE 0.31; all *p*_s_ < 0.013).

**FIGURE 3:**
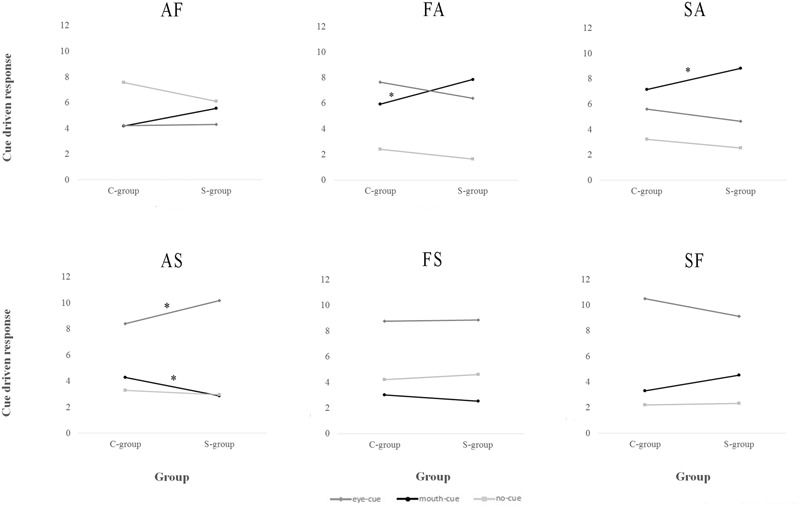
Number of Ebola Virus disease Survivor (S-group) and Controls (C-group) cue driven chimeric facial expressions recognitions displayed chimera by chimera. **(A)** AF, Anger_Fear chimera; **(B)** AS, Anger_Sadness chimera; **(C)** FA, Fear_Anger chimera; **(D)** FS, Fear_Sadness chimera; **(E)** SA, Sadness_Anger chimera; **(F)** SF, Sadness_Fear chimera. Only between-groups differences were shown. See the text for within-group differences. ^∗^*p* < 0.05. S-group, Survivor group; C-group, Control group.

These results can be better clarified considering the significant interaction Cue by Chimera by Group, indeed Sidak *post hoc* test conducted on this triple interaction revealed interesting differences between the two groups. Considering AF chimera (**Figure 3A**), S-group participants did not show significant differences in the use of the three cues in chimeric facial expression recognition (eye-cue: 4.32, SE 0.56; mouth-cue: 5.57, SE 0.59; no-cue: 6.11, SE 0.68; all *p*_s_ > 0.296). On the contrary, C-group participants’ chimeric facial expression recognition was significantly driven by no-cue, resulting no-cue significantly different from the other two cues (eye-cue: 4.23, SE 0.53; mouth-cue: 4.19, SE 0.56; no-cue: 7.58, SE 0.65; all *p*_s_ < 0.007). Comparing S-group’s and C-group’s performance during the recognition of the AF chimeric facial expressions, results showed the absence of significant between-groups differences (all *p*_s_ > 0.097).

Considering the AS chimera (**Figure 3B**), S-group participants’ chimeric facial expressions recognition was significantly driven by eye-cue, resulting eye-cue significantly different from the other two cues (eye-cue: 10.18, SE 0.51; mouth-cue: 2.86, SE 0.41; no-cue: 2.96, SE 0.43 all *p*_s_ < 0.000). Similarly, C-group participants’ chimeric facial expression recognition was significantly driven by eye-cue, resulting eye-cue significantly different from the other two cues (eye-cue: 8.42, SE 0.49; mouth-cue: 4.29, SE 0.39; no-cue: 3.29, SE 0.41; all *p*_s_ < 0.000). Comparing S-group’s and C-group’s performance during the recognition of the AS chimeric facial expressions, results showed that chimeric facial expressions recognition of S-group was driven more by eye-cue (*p* = 0.016) but less by mouth-cue (*p* = 0.014) than C-group.

Considering the FA chimera (**Figure 3C**), S-group participants’ chimeric facial expressions recognition was equally driven by eye-cue and mouth-cue, resulting these two significantly different from no-cue (eye-cue: 6.39, SE 0.58; mouth-cue: 7.86, SE 0.56; no-cue: 1.75, SE 0.41 all *p*_s_ < 0.000). Similarly, C-group participants’ chimeric facial expressions recognition was equally driven by eye-cue and mouth-cue, resulting these two significantly different from no-cue (eye-cue: 7.64, SE 0.55; mouth-cue: 5.93, SE 0.54; no-cue: 2.42, SE 0.39; all *p*_s_ < 0.000). Comparing S-group’s and C-group’s performance during the recognition of the FA chimeric facial expressions, results showed that chimeric facial expressions recognition of S-group was driven more by mouth-cue than C-group (*p* = 0.017).

Considering the FS chimera (**Figure 3D**), S-group participants’ chimeric facial expressions recognition was mostly driven by eye-cue, followed by no-cue and mouth-cue, resulting these three cues significantly different from each other (eye-cue: 8.86, SE 0.70; mouth-cue: 2.54, SE 0.41; no-cue: 4.61, SE 0.60; all *p*_s_ < 0.024). Differently, C-group participants’ chimeric facial expressions recognition was mostly driven by eye-cue, resulting this one significantly different from mouth-cue and no-cue (eye-cue: 8.77, SE 0.66; mouth-cue: 3.03, SE 0.39; no-cue: 4.19, SE 0.57; all *p*_s_ < 0.001). Comparing S-group’s and C-group’s performance during the recognition of the FS chimeric facial expressions, results showed the absence of significant between-group differences (all *p*_s_ > 0.387).

Regarding the SA chimera (**Figure 3E**), S-group participants’ chimeric facial expressions recognition was mostly driven by mouth-cue, followed by eye-cue and no-cue, resulting these three cues significantly different from each other (eye-cue: 4.64, SE 0.59; mouth-cue: 8.82, SE 0.57; no-cue: 2.54, SE 0.38; all *p*_s_ < 0.040). Differently, C-group participants’ chimeric facial expressions recognition was equally driven by eye-cue and mouth-cue, resulting these two significantly different from no-cue (eye-cue: 5.61, SE 0.56; mouth-cue: 7.16, SE 0.54; no-cue: 3.23, SE 0.37; all *p*_s_ < 0.011). Comparing S-group’s and C-group’s performance during the recognition of the SA chimeric facial expressions, results showed that chimeric facial expressions recognition of S-group was driven more by mouth-cue than C-group (*p* = 0.038).

Regarding the SF chimera (**Figure 3F**), S-group participants’ chimeric facial expressions recognition was mostly driven by eye-cue, followed by mouth-cue and no-cue, resulting these three cues significantly different from each other (eye-cue: 9.14, SE 0.71; mouth-cue: 4.54, SE 0.62; no-cue: 2.32, SE 0.45; all *p*_s_ < 0.024). Differently, C-group participants’ chimeric facial expressions recognition was mostly driven by eye-cue, with no significant difference between mouth-cue and no-cue (eye-cue: 10.52, SE 0.67; mouth-cue: 3.30, SE 0.58; no-cue: 2.19, SE 0.42; all *p*_s_ < 0.000). Comparing S-group’s and C-group’s performance during the recognition of the SF chimeric facial expressions, results showed the absence of significant between-group differences (all *p*_s_ > 0.148).

## Discussion

The recognition of social signals, such as facial expressions of emotions, is an important developmental ability that can be altered by the exposure to traumatic experiences during childhood. The aim of the present study was to investigate if the bias in the explicit recognition of the facial expression of anger can be described as a victims’ overall response tendency toward anger recognition or whether specific facial expressive cues of anger became more salient after trauma exposure. To solve this question, a group of underage Sierra Leonean Ebola survivors and a control group performed a forced-choice recognition task of chimeric facial expressions of emotions in which upper and lower half faces displaying different negative emotions (i.e., anger, fear, and sadness) were paired. Furthermore, to assess the principal psychological and psychiatric sequelae of trauma exposure, for the first time three clinical questionnaires (i.e., IES-R, PCL-5 and CERQ-short) were translated in Krio, the Sierra Leonean de facto national language, and tested.

Ebola survivors showed higher presence of PTSD related symptoms – as evaluated by the IES-R and PCL-5 questionnaires – with respect to controls. Emotion regulation in adaptation to stressful life events was assessed by the CERQ-short questionnaire demonstrating that Ebola survivors showed higher incidence of rumination and catastrophizing tendencies but also greater refocus on planning and positive reappraisal coping strategies with respect to controls. These results confirm the traumatic nature of Ebola infection and related adversities (e.g., family members’ death, hospitalization, and subsequent stigmatization) able to induce PTSD-related sequelae and specific coping strategies among Ebola survivors. The two groups shared the same sociocultural background and came from the same area in Sierra Leon, consequently they were both exposed to Ebola outbreak but at a different degree of impact. Only Survivor group participants were infected by Ebola Virus and lost parents and close relatives during the outbreak, whereas Control group participants, thanks to the prompt dislocation and institution of sanitary check points, were not infected and did not experience mourning directly related to Ebola outbreak.

To the best of our knowledge, this is the first time that PTSD diagnostic questionnaires and coping strategies scale were adapted, translated and applied in underage Sierra Leonean population exposed to traumatic events. A similar procedure was followed by previous studies that translated and adapted scales investigating PTSD-related symptoms and other psychiatric and psychological sequelae (i.e., major depression and anxiety) in West-African adult populations (Bolton et al., 2002; Johnson et al., 2008; Betancourt et al., 2010, 2011, 2016). Results demonstrate that the socio-cultural adaptation procedure followed in the present study designs sensible scales able to highlight the effect of early acute traumatic experiences in underage people even when they were exposed to different degrees of impact of the same traumatic event.

Considering participants’ performance with the forced-choice recognition task of chimeric facial expressions of emotions, regardless of group membership, all participants tended to recognize the chimeric facial expressions taking advantage from the upper facial expressive cues with respect to the lower ones. This result is coherent with part-based chimeric facial expression processing according to which, particularly for the facial expression of anger, fear and sadness, the eye-region is the most significant for the identification of the new configural chimeric facial expressions (Calder et al., 2000). The relevance of the eye-region for the correct identification of negative facial expressions like anger, fear and sadness is extensively established (Kestenbaum, 1992; Kohler et al., 2004; Eisenbarth and Alpers, 2011; Guo, 2012; Gagnon et al., 2014; Schurgin et al., 2014; Wells et al., 2016; Elsherif et al., 2017) even if not always replicated, likely due to differences in the methodology and technique involved (see for example, Kotsia et al., 2008).

Focusing on the main aim of the present study, groups’ performance at the forced-choice recognition task of chimeric facial expressions of emotions demonstrated that the bias in the recognition of angry facial expressions does not reflect a mere response tendency toward anger recognition but rather a specific greater salience of facial expressive cues of anger. This conclusion is supported by two main sources of evidence. First, the recognition of chimeric facial expressions not composed by facial expressive cues of anger (i.e., FS, SF chimeras) was mostly driven by eye-cue with no between-groups difference. This means that in the absence of angry facial expressive cues, participants’ identification performance was not determined by a general recognition tendency toward anger of the traumatized population, but by the emotional expression derived by the upper half-face of the chimeric facial expression, in agreement with the part-based analyses processing. Second, when participants were asked to recognize chimeric facial expressions composed by facial expressive cues of anger, the two groups showed different patterns of response. Specifically, when participants were involved in the recognition of chimeric facial expressions composed by angry and sad expressive cues (i.e., AS, SA chimeras), S-group explicitly recognized the chimeric facial expressions using more than the C-group the angry facial expressive cues, regardless of their position. In other words, S-group used more the eye-cue and the mouth-cue than C-group during the recognition of the AS and SA chimeras, respectively. A similar response pattern was shown during the recognition of the FA chimera, where S-group’s recognition performance was driven more by mouth-cue than the C-group. These response patterns clearly point to the unbalanced salience of facial expressive cues of anger. Indeed, both angry eye-cue and mouth-cue acquired greater perceptive salience after trauma exposure, steering Ebola victims’ explicit recognition of chimeric facial expressions more than controls. These results demonstrate that facial expressive cues of anger, when present, do influence S-group’s identification performance.

Unexpectedly, the greater salience of facial expressive cues of anger was not demonstrated during the recognition of AF chimeras. One explanation could be that this specific condition facilitates a holistic face processing rather than a part-based identification processing as the one shown with the other chimeras. The expected and previously demonstrated part-based analyses of the upper and lower half facial expressions was not evoked, giving way to a completely configurational analysis of the overall chimeric facial expression, as revealed by participants’ response strategies. S-group participants did not show a clear preference for any of the cues (i.e., eye-cue, mouth-cue, and no-cue), in fact they solved the forced-choice recognition task by applying a “gamble response strategy,” following which each response alternative was chosen with the same probability. Differently, C-group participants selected significantly more frequently the no-cue response instead of both eye-cue and mouth-cue alternatives, showing a sort of “radical third way strategy.” The absence of a clear dominance of one of the two half-faces induced different holistic response strategies in the two groups, but in both cases, it did not reveal a bias in the recognition facial expressive cues of anger. The manifestation of these two different response strategies was due to the specific interaction between the nature of the facial expressions (i.e., anger and fear) and the position of the expressive cues showed (i.e., angry eye-cue and fearful mouth-cue): in fact, this response pattern was visible only during the recognition of AF chimeric facial expressions. Even if, as mentioned before, the literature is coherent in attributing to the eye region of both angry and fearful faces the highest saliency in the identification of the overall facial expressions, it can be useful to highlight here some peripheral phenomena that can account for this unexpected result. Kohler et al. (2004) found that in every facial expression tested (i.e., sadness, happiness, and fear), except anger, the opening of the mouth, as represented by parted lips or dropped jaw (i.e., the prototypical mouth feature of fearful facial expressions), correlated with improved recognition. Furthermore, Schurgin et al. (2014), using the images obtained by the same database adopted in the present study, demonstrated that the occlusion of the eye region altered the effective recognition of the facial expressions of anger but not of those expressing fear. Even if this evidence cannot exhaustively explain the present result, it points out the specific interplay between angry eyes and fearful mouth, likely facilitating the holistic analysis of this specific chimeric facial expression.

Taken together, the present results demonstrate that the bias in the recognition of facial expressions of anger does not reflect a mere response tendency toward anger recognition but rather a specific greater salience of facial expressive cues of anger. Indeed, only when they were present (with the exception of the AF chimera), the recognition of chimeric facial expressions in the S-group was more strongly biased in favor of angry expressive cues than in the C-group, regardless of their position in the chimeric facial expression. The present results suggest that trauma exposure during childhood does not affect victims’ overall emotion recognition abilities, but it specifically induces alterations in the perceptual mechanisms involved in the processing of phylogenetically inherited motor patterns, like facial expressions. Being exposed to traumatic events during childhood can lead victims to filter and select some environmental cues at the expense of others, leading to an unequal processing of external information and a biased recognition of others’ facial expressions of emotion. These results are coherent with previous studies suggesting that early traumatic experiences affect the developing perceptual systems, in part by lowering the sensory threshold for anger-related to recruit the attentional focus (Pollak, 2003). Several studies demonstrated both at the behavioral (Pollak and Tolley-Schell, 2003) and electrophysiological level (Pollak and Cicchetti, 1997; Pollak et al., 2001; Cicchetti and Curtis, 2005; Shackman et al., 2007; Curtis and Cicchetti, 2011) that victims of abuses and maltreatments during childhood show greater attentional focus to angry faces regardless of their contingent relevance. Relatedly, the attentional bias to angry faces was shown to be associated with anxiety individual traits (Derryberry and Reed, 2002; Fox et al., 2002).

The present results acquire greater importance by considering the interplay between evolutionary adaptive purposes and basic emotions as response-coordination ‘packages’ shaped by evolution to meet particular environmental challenges (Ekman, 1992). At a phylogenetic level, one of the most important evolutionary challenge is the rapid, effective and discerning facial expressions of emotions recognition, inasmuch it allows coherent and adaptive behavioral reactions in agreement with the emotional content of facial expressions. According to BET theory, it suggests that the inherited motor pattern associated to the facial expression of emotion is the optimal facial configuration resulted from the interaction between several evolutionary demands which include the effective recognition of facial expression of emotion. This consideration can justify the role of multiple facial features and their inter-relationship in the discrimination of different facial expressions. At an ontogenetic level, the exposure to hostile and negative environment tunes the perceptual and attentional mechanisms involved in the recognition of facial expressions of emotions exacerbating the saliency of specific motor patterns conveying anger. In other words, the environment selects both the most effective facial motor pattern and the perceptive and attentive salience attributed to specific expressive facial cues influencing not only the expression but even the recognition of basic facial expressions of emotions.

Some limitations of the present study need to be highlighted. First, to avoid possible confounding effects, two populations sharing the same sociocultural background were involved in the study. Consequently, even if S-group and C-group clearly differ in the direct impact of the traumatic event here considered as demonstrated by the scoring obtained with clinical scales, both groups of participants were exposed to the Ebola outbreak. This circumstance might have partially reduced between-groups differences. Second, participants’ scarce familiarity with computers prevented the collection of reaction times during the recognition task of chimeric facial expressions, which could be a useful variable to better understand the bias in the recognition of facial expressions of anger (Calder et al., 2000). Finally, to accomplish the main aim of the present study, only chimeric facial expressions were employed. In order to better understand the perceptual and attentive inspection modalities of real facial expressive cues, further studies should investigate victims’ visual scan-path during facial expression recognition.

## Conclusion

The present study addressed an important and not yet completely clarified issue about the mechanisms underlying the bias in the recognition of facial expressions of anger following childhood trauma exposure. Results demonstrate that the perceptual salience of both eye and mouth cues of angry facial expressions increases among victims of childhood trauma. Being exposed to early adverse and negative experiences, rather than producing an overall response tendency toward anger recognition, tunes the perceptual analysis of angry facial expressive cues, leading to the explicit biased recognition of emotions.

## Author Contributions

MA designed the study, collected, analyzed and interpreted the data, finally she wrote the manuscript. VE was principally engaged in participants’ recruitment and data collection, furthermore she gave her contribution to results interpretation. VE supervised the cross-cultural adaptation of the clinical scales employed in the present study. FF participated to data analyses and contributed to the drafting of the manuscript. MU, VG, and RR designed the study, interpreted the data and drafted the manuscript. All authors approved the final version of the manuscript.

## Conflict of Interest Statement

The authors declare that the research was conducted in the absence of any commercial or financial relationships that could be construed as a potential conflict of interest.
