# Supplementary material for: Evidence for Anger Saliency during the Recognition of Chimeric Facial Expressions of Emotions in Underage Ebola Survivors
Source: Front Psychol. 2017 Jun 23;8:1026. doi: 10.3389/fpsyg.2017.01026 (PMC5482096; doi:10.3389/fpsyg.2017.01026)
Supplement: Supplementary file 1 [file Data_Sheet_1.DOCX]

Supplementary Material

**Evidence for anger saliency during the recognition of chimeric facial expressions of emotions in underage Ebola survivors.**

**Martina Ardizzi*, Valentina Evangelista, Francesca Ferroni, Maria Alessandra Umiltà, Roberto Ravera and Vittorio Gallese**

*** Correspondence:** Corresponding Author: [martina.ardizzi@unipr.it](mailto:martina.ardizzi@unipr.it)

# Supplementary Data

**Cross-Cultural Adaptation procedure**

The English version of the three clinical scales (PCL-5, Weathers et al., 2013; IES-R, Weiss and Marmar, 1996; CERQ-short, Garnefski and Kraaij, 2006) was submitted to the Process of Cross-Cultural Adaptation of Self-Report Measures following the guidelines (Beaton et al., 2000). This process provides not only an accurate translation from the original language to the local language but it also assesses the cultural and social relevance of the translated scales according to the target population. The cross-cultural adaptation procedure was implemented on both instruction statements, items and response options.

The cross-cultural process encompasses 5 stages described hereunder:

Stage I: Forward Translation

Two forward translations were made from the original language (i.e., English language) to the target language (i.e., Krio language) by two bilingual translators whose native language was the target language and who were highly fluent in English. The two translators had different professional profiles and backgrounds. The first translator was aware of the concepts being examined in the questionnaires in order to assure equivalence from a clinical perspective. The second translator was neither aware nor informed of the concepts being quantified by the questionnaires and had no medical or clinical background. The inclusion of a naive translator less influenced by the clinical issues offered a translation more reflecting the language used by that population, highlighting ambiguous meanings in the original questionnaires. The two translations were accompanied by written explicative reports listing additional comments to highlight possible challenging sentences or uncertainties. The rationale followed for the translation choices was also summarized in the written report.

Stage II: Synthesis of the translations

The two translators and a local supervisor summarized the first translator’s (T1) and the second translator’s (T2) versions in a common form (T12). A written report carefully documenting the synthesis process was drawn up. The final version was drafted in Krio by using English alphabet instead of Krio alphabet because it is currently the most used one.

Stage III: Back translation

From the T12 version, two back translations (T3, T4) were obtained by two independent translators whose native language was English and who had high competence in Krio language. The translators were totally blind to the original version and to the concepts explored by the scales, and they had not medical or psychological background. This stage of the cross-cultural adaptation process can highlight inconsistencies, erroneous interpretations and unexpected meanings in the target version of the questionnaires.

Stage IV: Expert Committee and final version

An expert committee including the translators involved in the forward and back translations and the local supervisor took part to the stage IV of the cross-cultural adaptation process. The aim of this phase was to validate and ensure the cross-cultural equivalence of the translated version of the scales. The semantic equivalence, cultural suitability and linguistic intelligibility of the instruction statements, items and response options of the questionnaires were evaluated by the expert committee through a 7-point Likert scale. The statements receiving a score below 7 were discussed, collegially changed and then re-submitted to the committee judgment. For the PCL-5 scale, 3 statements were initially judged below the threshold. Considering the IES-R questionnaire, 4 statements were initially judged below the threshold. Finally, for the CERQ scale, 3 statements were initially judged below the threshold. Finally, the committee sentenced that the final version was “easy to understand and appropriate to the Sierra Leonean cultural context”.

Stage V: Test the final version

The final stage of cross-cultural adaptation process was the pre-test. The adapted and translated versions of the questionnaires were submitted to a sample of 20 schooled individuals recruited in the Freetown urban area, with middle income and not included in the main experiment (Test-group; mean age 21.25 years, SE 7.7; 13 males). The mean scores obtained at each questionnaire and relative subscales are listed in the table below (see Table S1).

Each participant completed the questionnaires and a report about scales intelligibility in which both the meaning of items and responses were explored. No participant described difficulties in understanding the questionnaire instructions and items and in the formulation of spontaneous responses.

# Supplementary Table

| **Questionnaire** | **Subscales** | **Test-group score** |
| --- | --- | --- |
| IES_TOT |  | 0.09; SE 0.09 |
|  | Intrusion | 0.11; SE 0.11 |
|  | Avoidance | 0.06; SE 0.06 |
|  | Hyperarousal | 0.08; SE 0.08 |
| PCL5_TOT |  | 18.20; SE 3.53 |
|  | Cluster B | 6.30; SE 1.19 |
|  | Cluster C | 2.50; SE 0.49 |
|  | Cluster D | 5.00; SE 1.28 |
|  | Cluster E | 4.40; SE 0.95 |
| CERQ |  |  |
|  | Self Blame | 3.10; SE 0.31 |
|  | Acceptance | 3.47; SE 0.14 |
|  | Rumination | 5.79; SE 0.35 |
|  | Positive Refocusing | 6.84; SE 0.47 |
|  | Refocus on planning | 7.10; SE 0.46 |
|  | Positive reappraisal | 6.74; SE 0.53 |
|  | Putting in to perspective | 5.84; SE 0.47 |
|  | Catastrophing | 5.58 SE 0.41 |
|  | Other blame | 4.00; SE 0.49 |
|  |  |  |

**Supplementary Table 1.** Questionnaire scores of Test group
