# Supplementary figures and images for: Evidence for Anger Saliency during the Recognition of Chimeric Facial Expressions of Emotions in Underage Ebola Survivors
Source: Front Psychol. 2017 Jun 23;8:1026. doi: 10.3389/fpsyg.2017.01026 (PMC5482096; doi:10.3389/fpsyg.2017.01026)

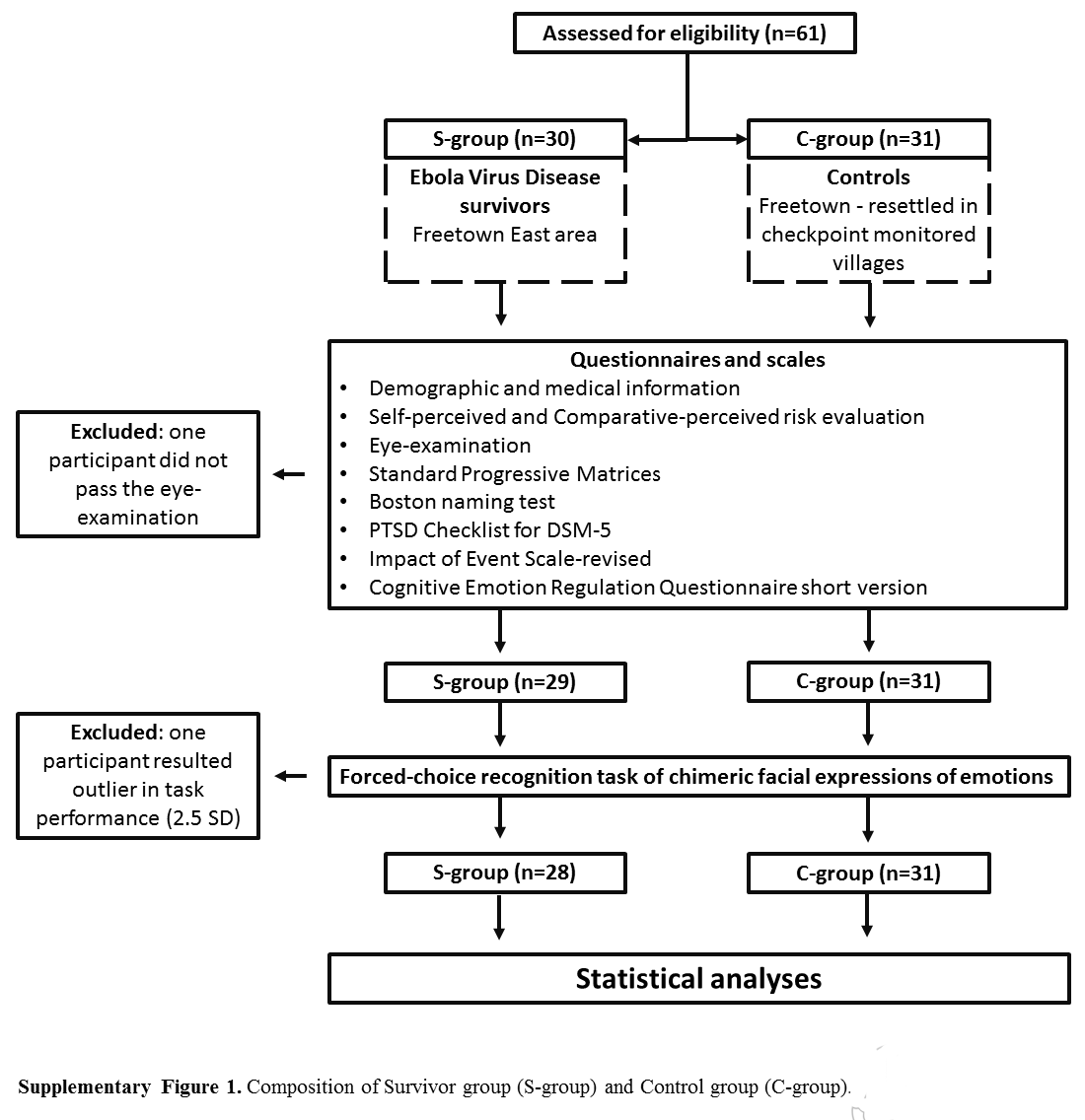

Supplement: Supplementary file 2 [file Image_1.tif]
